# Circular RNA expression in turkey skeletal muscle satellite cells is significantly altered by thermal challenge

**DOI:** 10.3389/fphys.2024.1476487

**Published:** 2024-09-18

**Authors:** Ashley A. Powell, Sandra G. Velleman, Gale M. Strasburg, Juan E. Abrahante Lloréns, Kent M. Reed

**Affiliations:** ^1^ Department of Veterinary and Biomedical Sciences, University of Minnesota, St. Paul, MN, United States; ^2^ Department of Animal Sciences, The Ohio State University, Wooster, OH, United States; ^3^ Department of Food Science and Human Nutrition, Michigan State University, East Lansing, MI, United States; ^4^ Minnesota Supercomputing Institute, University of Minnesota, Minneapolis, MN, United States

**Keywords:** non-coding RNA, circRNA, *Meleagris gallopavo*, *Pectoralis major*, differential splicing

## Abstract

**Introduction:**

Understanding the genetic mechanisms behind muscle growth and development is crucial for improving the efficiency of animal protein production. Recent poultry studies have identified genes related to muscle development and explored how environmental stressors, such as temperature extremes, affect protein production and meat quality. Non-coding RNAs, including circular RNAs (circRNAs), play crucial roles in modulating gene expression and regulating the translation of mRNAs into proteins. This study examined circRNA expression in turkey skeletal muscle stem cells under thermal stress. The objectives were to identify and quantify circRNAs, assess circRNA abundance following RNAse R depletion, identify differentially expressed circRNAs (DECs), and predict potential microRNA (miRNA) targets for DECs and their associated genes.

**Materials and methods:**

Cultured cells from two genetic lines (Nicholas commercial turkey and The Ohio State Random Bred Control 2) under three thermal treatments: cold (33°C), control (38°C), and hot (43°C) were compared at both the proliferation and differentiation stages. CircRNA prediction and differential expression and splicing analyses were conducted using the CIRIquant pipeline for both the untreated and RNase R depletion treated libraries. Predicted interactions between DECs and miRNAs, as well as the potential impact of circRNA secondary structure on these interactions, were investigated.

**Results:**

A total of 11,125 circRNAs were predicted within the treatment groups, between both untreated and RNase R treated libraries. Differential expression analyses indicated that circRNA expression was significantly altered by thermal treatments and the genetic background of the stem cells. A total of 140 DECs were identified across the treatment comparisons. In general, more DECs within temperature treatment comparisons were identified in the proliferation stage and more DECs within genetic line comparisons were identified in the differentiation stage.

**Discussion:**

This study highlights the significant impact of environmental stressors on non-coding RNAs and their role in gene regulation. Elucidating the role of non-coding RNAs in gene regulation can help further our understanding of muscle development and poultry production, underscoring the broader implications of this research for enhancing animal protein production efficiency.

## 1 Introduction

Animal agriculture plays an important role in meeting human nutritional needs, primarily by providing complex muscle proteins as meat. Currently, poultry is the largest sector of meat consumed in the US, with consumption levels continuing to rise. Turkey meat accounts for approximately 10% of the poultry meat revenue in the U.S. (United States Department of Agriculture- NASS, 2022). Sustainability in our food systems is crucial for ensuring long-term food security, reducing environmental impact, and promoting the health and wellbeing of current and future generations. Efficient production of animal protein is key to sustainable agriculture and successfully meeting meat consumption demands. Efficient *Pectoralis major* muscle (breast meat) production and growth performance has been and continues to be a focus of turkey breeding efforts (Duggan et al., 2023; Musselman, 2021).

Understanding the underlying genes involved in muscle meat growth and development can help further efforts towards increasing the efficiency of animal protein production. Recently, studies in poultry have identified genes and other factors related to muscle development and have begun to gather a better understanding of how environmental stressors can affect protein production and meat quality. Temperature extremes are one source of environmental stress that has detrimental effects on poultry production including increased fat deposition, compromised proteins, reduced growth rates, and even the death of young birds (Barnes et al., 2019; Henrikson et al., 2018; Lara and Rostagno, 2013; Nawab et al., 2018). Transcriptomics research has revealed genes (e.g., myostatin) and gene pathways (e.g., lipid deposition and growth hormone-boosting peptide pathways) involved in muscle development and meat quality (Dou et al., 2018; Ibrahim et al., 2021; Li et al., 2024). Identifying key genes and understanding their expression patterns reveals only part of the complex system that controls overall gene expression. Non-coding RNAs, such as long non-coding RNAs (lncRNAs) and micro RNAs (miRNAs), have been shown to play important roles in modulating gene expression through binding of DNA, mRNA, and proteins (O’Brien et al., 2018; Statello et al., 2021).

Growing evidence suggests that other classes of non-coding RNAs, such as circular RNAs (circRNAs), also play crucial roles in the regulation of gene expression (Lei et al., 2022; Reed et al., 2021; Tian et al., 2023; Wilusz, 2018). These single-stranded, closed-loop, non-coding RNAs are formed by a type of alternative splicing known as backsplicing, where the 3′ end of an exon covalently bonds to the 5′ end of an upstream exon. Several functions of circRNAs are hypothesized, including acting as molecular “sponges” that bind cellular miRNAs, serving as transcriptional regulators, and potentially acting as templates for translation into peptides (Barrett and Salzman, 2016; Tian et al., 2023; Wilusz, 2018; Zucko and Boris-Lawrie, 2020). Conservation of function of circRNAs is still being explored with many studies showing circRNA expression and function to be species specific (Santos-Rodriguez, Voineagu, and Weatheritt, 2021; Li and Wang, 2024) while other studies show conservation of circRNAs across species and even kingdoms (Gao et al., 2020; Huo et al., 2018), therefore predicting function of a circRNA can be limited by genome annotations within a given species. Previous studies have indicated that circRNAs may be crucial in muscle development and thermal stress responses (Ouyang et al., 2018; Reed et al., 2021).

Historically, circRNAs have been difficult to study due to a lack of bioinformatics resources. Until recently, distinguishing between linear and circular RNAs was difficult, but new tools have emerged for data-mining these specific RNA structures in RNAseq datasets (Barrett and Salzman, 2016; Gao, Wang, and Zhao, 2015; Wu et al., 2019). Multiple new tools utilizing different algorithms and identification approaches have been developed (Vromman et al., 2023), including the CIRI program and pipelines that accurately identify complementary backsplice junctions (Gao, Wang, and Zhao, 2015; Zhang et al., 2020).

In a previous study, we explored circRNAs as a novel facet of gene regulation and identified significant differential expression of circRNAs in thermally challenged turkey (*Meleagris gallopavo*) poults from different genetic lines (Reed et al., 2021). The first week post-hatch is critical for muscle development, as muscle stem cells (satellite cells, SCs) exhibit peak mitotic activity and are highly sensitive to temperature changes during this timeframe (Halevy et al., 2000; Mozdziak, Schultz, and Cassens, 1994; Patael et al., 2019; Piestun et al., 2017; Xu et al., 2021). Thermal stress during this period may alter the characteristics of SCs and result in changes in *Pectoralis major* muscle development.

In this study, we employed a data-mining approach to investigate differential expression of circRNAs in cultured turkey *Pectoralis major* muscle satellite cells (SCs) between and within SCs isolated from a modern commercial turkey and a historic slower growing turkey line under different thermal treatments. This investigation had four main objectives: 1) Identify and quantify circRNAs; 2) Test circRNA abundance in RNA following RNAse R depletion; 3) Identify differentially expressed circRNAs (DECs); and 4) Predict potential miRNA targets for DECs and their associated genes. Our long-term goal is to better understand the role of non-coding RNAs in gene regulation, specifically in the context of thermal stress responses during muscle development and its implications for poultry production.

## 2 Materials and methods

### 2.1 Materials

Transcript sequence data from the turkey SC libraries previously described in Reed et al. (2022) were used for circRNA analyses. Data are accessioned as part of SRA BioProject PRJNA842679. For the purposes of this study, the experimental lines, thermal treatments, and data management are summarized below.

The control line (Randombred Control Line 2, RBC2), is a turkey line established in 1966 from commercial birds of that time and has been maintained at the Poultry Research Center at The Ohio State University (Nestor, 1977) without any conscious selection. The experimental line (Nicholas Commercial Turkeys, NCT), is a commercial meat-type turkey line selected for performance (yield, weight gain, feed conversion, etc.) in a modern turkey production system (Aviagen^®^ Turkeys, Inc.). Cultured 1-week cells from both lines were subjected to proliferation and differentiation protocols to create four sets of SCs to be used in experimental treatments: RBC2-proliferation (RBC2-pro), RBC2-differentiation (RBC2-dif), NCT-proliferation (NCT-pro), and NCT-differentiation (NCT-dif). The satellite cell (SC) harvest, maintenance, differentiation and proliferation assays, and sequence data handling were previously described in detail in Reed et al. (2017), Reed et al. (2022), Reed et al. (2023). Three thermal treatments were assigned to each SC set: 33°C (cold), 38°C (control), and 43°C (hot). The RBC2-pro-cold treatment resulted in poor cell growth and RNA recovery and this treatment was excluded from sequencing. The remaining 11 experimental SC groups were replicated creating 22 SC libraries available for sequencing and analysis. Data from an analysis of small RNA (miRNA) sequencing from the same biosources (Reed et al., 2023) were also available for comparative analyses.

The reference turkey genome assembly and annotation (UMD5.1) from Ensembl (Meleagris_gallopavo.Turkey_5.1. dna.toplevel.fa genome; Meleagris_gallopavo.Turkey_5.1.109. gtf) were used to conduct these analyses.

### 2.2 Methods

#### 2.2.1 circRNA predictions and quantifications

Prediction and quantification of circRNAs was performed with the python-based circRNA quantification and differential expression program: CIRIquant (Zhang et al., 2020). CircRNA identification was performed with the CIRI2 program that is embedded within CIRIquant. CIRI2 predicts circRNAs based on an algorithm that detects and differentiates between back-spliced junction (BSJ) reads and non-BSJ reads to reduce the number of false positives with a high level of sensitivity (Gao et al., 2015). Quantification was performed via CIRIquant and by re-alignment of RNA-seq reads against a pseudo-circular reference. Abundance of circRNA was estimated by comparison of BSJ reads and front-spliced reads (FSJ).

#### 2.2.2 RNase depletion study

As a partial verification of circRNA prediction, and to assess depletion protocols for future circRNA studies, RNA of the SCs from the NCT control temperature group for both proliferation (NCT-pro-R) and differentiation (NCT-dif-R) was subjected to an RNase depletion protocol. Briefly, RNA samples were treated with RNase R enzyme to digest linear RNA molecules while retaining lariat or circular RNA structures. Digestion was performed on 4 ug of total DNAse-treated RNA using 10 units of RNase R exoribonuclease (Lucigen, Corp) following manufacturer’s protocol (incubation reaction at 37°C for 20 min followed by 65°C for 20 min). The resulting RNAs were quantified and submitted to the University of Minnesota Genomics Center for library preparation and sequencing. The libraries were sequenced on the Illumina NovaSeq SP platform. Prediction of circRNAs in depleted samples was performed with CIRIquant utilizing the “RNase R effect correction” function. The “RNase R effect correction” function uses both the depleted and un-depleted expression to estimate the before-treatment expression levels of circRNAs detected in RNase R data (Zhang et al., 2020).

#### 2.2.3 Differential expression analyses

##### 2.2.3.1 Differential expression

Differential expression analyses were conducted with CIRIquant (Zhang et al., 2020) and differentially expressed circRNAs (DECs) in each comparison were identified as having FDR *p*-value <0.05 and log fold change (LogFC) > 1.0. DECs were predicted from the CIRIquant option that allowed for biological replicates (comparison was on treatment vs. treatment basis).

##### 2.2.3.2 Differential circRNA splicing (circRNA vs. linear forms)

Differential splicing analyses were conducted with CIRIquant, using the option that did not allow for biological replicates (comparison was on a sample vs. sample basis). The likelihood of an RNA being found in its linear *versus* circular form is defined by the differential splicing (DS) score (Zhang et al., 2020). Splicing scores of DECs previously identified were averaged across sample comparisons for a final average DS score.

#### 2.2.4 Functional prediction analyses

##### 2.2.4.1 circRNA/miRNA interaction prediction

Potential interactions between the DECs and turkey micro-RNAs (miRNAs) were investigated through target binding site predictions. Targets were predicted by aligning miRNAs identified in turkey skeletal muscle satellite cells (Reed et al., 2023) with the sequences of the DECs with miRanda v3.3a (Enright et al., 2003) using position-weighted scoring, alignment score of >165, and |energy-kcal/mol| >7.0.

##### 2.2.4.2 Secondary structure prediction

To investigate the effect of secondary structure on circRNAs, RNAfold (Lorenz et al., 2011) was used to examine a circRNA and its ability to bind with its associated miRNAs. Minimum free energy (MFE) and the thermodynamic ensemble (TE) structures were predicted at 37°C, 38°C [approximate temperature of newly hatched poults (Strasburg, unpublished) and control temperature for the cultured SC cells], and 39°C for circ09021. This circRNA was chosen based upon nucleotide (nt) length limits of RNAfold (10,000 nt) and had corresponding miRNA targets. This allowed assessment of TE structure at different temperatures and binding access of miRNAs to the targets predicted in the circRNA sequence. The associated miRNAs (alignment score of >150) were then aligned with the predicted secondary structure to assess if temperature affected binding ability.

## 3 Results

### 3.1 circRNA predictions and quantifications

**FIGURE 1:**
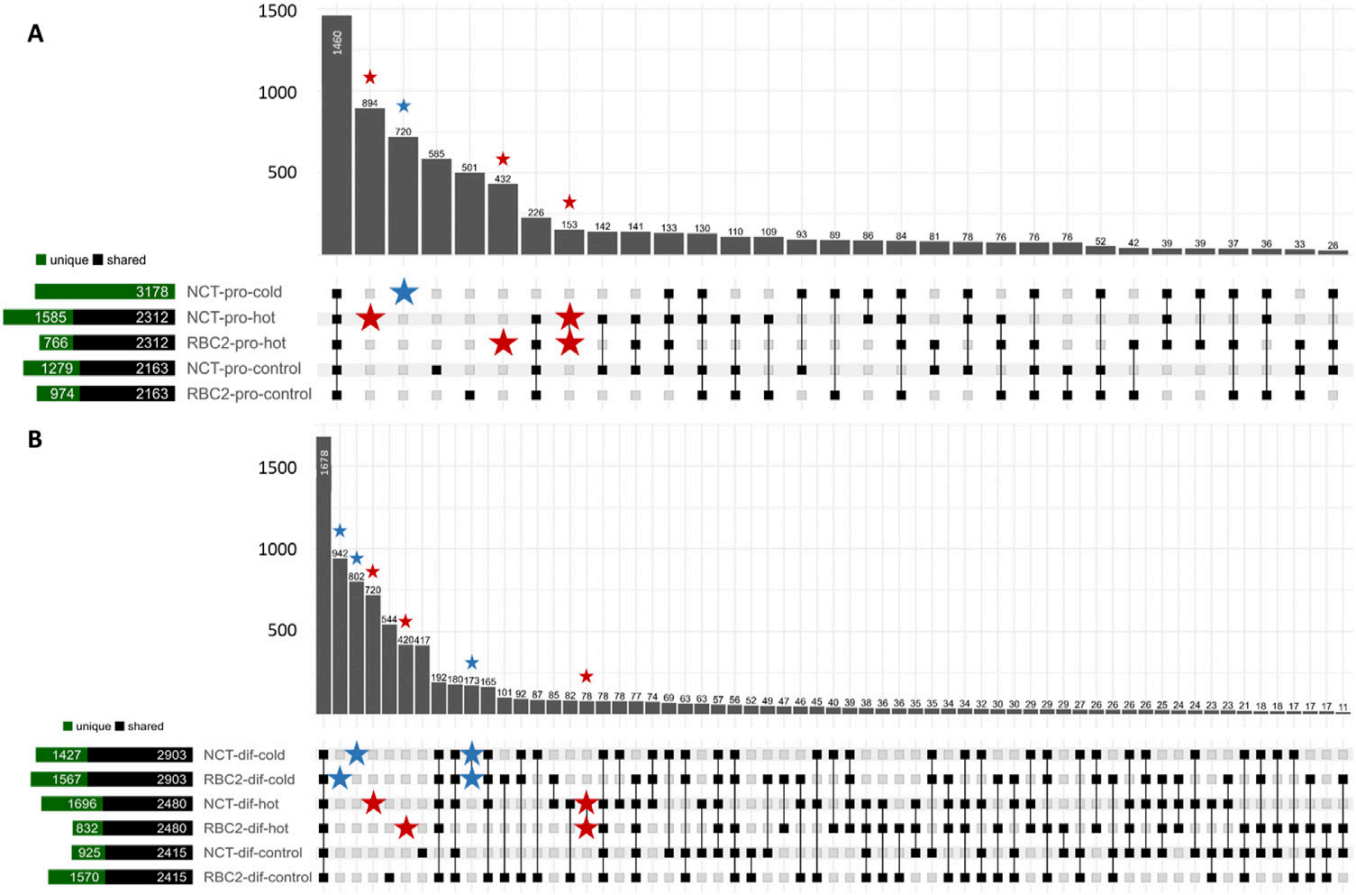
Upset plot depicting circRNAs predicted in the SCs in the proliferation stage **(A)** and the differentiation stage **(B)** by treatment. Set size bar plot shows circRNAs compared between RBC2 and NCT treatment equivalents (i.e., RBC2-pro-control vs. NCT-pro-control) to depict circRNAs shared between or unique to the treatments. circRNAs predicted only in cold treatments are marked with a blue star and circRNAs predicted only in hot treatments are marked with a red star.

A total of 10,679 unique circRNAs, mapping across 76 chromosomes and genome contigs, was identified across the 22 non-depleted SC libraries. In these non-depleted samples, 517 circRNAs were common and observed in all libraries, whereas 446 circRNAs were unique and only observed in single libraries (Figure 1; Supplementary Table S1). When combining libraries by treatment and sample type, 1,460 circRNAs were predicted to occur across all treatments and 3,132 circRNAs were unique to single treatments.

A total of 7,344 circRNAs was observed in the RBC2 SCs (non-depleted libraries), and 8,363 in the NCT SCs with 2,316 and 3,335 circRNAs unique to the RBC2 and NCT libraries, respectively. A greater number of circRNAs were predicted in SCs from the proliferating cells (6,779) than differentiating cells (5,198), with 4,847 and 3,266 circRNAs unique to SCs in proliferation and differentiation, respectively. When segregated by temperature treatment, 6,488 circRNAs were observed in the heat-treated SCs (43°C), 6,725 circRNAs were observed in the cold-treated SCs (33°C), and 6,334 were observed in the control treatment (38°C), with 1810, 2039, and 1,581 circRNAs unique to the hot, cold and control-treatment cells, respectively.

### 3.2 RNase depletion study

Analysis of sequences from the RNase depletion libraries revealed 792 circRNAs not previously detected in the non-depleted counterparts. In total, 446 novel circRNAs were predicted across the depleted proliferation and differentiation libraries (Supplementary Table S2). In the NCT-pro RNase comparison, 190 circRNAs underwent a quantification correction by CIRIquant when the RNase R effect correction was applied (Table 1). In the NCT-dif RNase R comparison, 712 circRNAs underwent a correction by CIRIquant with an additional 6 circRNAs being predicted in the CIRIquant adjusted library only and 3 circRNAs no longer being detected (Table 1).

**TABLE 1:** Comparison of circRNAs predicted in NCT control libraries, their RNase depleted counterpart libraries, and the CIRIquant RNase R effect correction output.

SC library	Total^a^	Unique^b^	Adjusted/Add/Loss^c^
NCT-pro-control	2,712	2,365	*NA*
NCT-pro-R	523	176	*NA*
NCT-pro-adjusted	2,888	0	14/176/0
NCT-dif-control	2,469	1920	*NA*
NCT-dif-R	1,216	667	*NA*
NCT-dif-adjusted	3,142	6	36/673/3

^a^ total number of circRNAs, predicted in each library.

^b^ number of circRNAs, that are unique to that library when compared to its counterpart or the adjusted library compared to both the un-depleted and depleted sample counterpart libraries.

^c^ number of circRNAs, in the “adjusted” sample libraries that have predicted circRNA, scores corrected by CIRIquant, number of circRNAs, that are unique to the “adjusted” sample libraries as determined by CIRIquant, and the number of circRNAs, no longer detected in the “adjusted” libraries as determined by CIRIquant.

### 3.3 Differential expression analyses

#### 3.3.1 Differential expression

Twelve expression comparisons were made between SC lines and temperature treatments. Across all comparisons, a total of 292 instances of significant differential expression involving 140 circRNAs, were found with an average of 22 circRNAs (range: 8–35) differentially expressed in each treatment comparison. Length of these circRNAs ranged from 180 to 115,731 nt, with an average length of 31,280 nt. Results for each treatment comparison are summarized in Table 2 and Supplementary Table S3. A complete list of genes associated with each DEC can be found in Supplementary Table S4.

**TABLE 2:** Summary of differentially expressed circRNAs by treatment.

Treatment comparison^a^	Up^b^	Down^c^	Total	DS Up^d^	DS down^e^	# miRNA tbs^f^
*Proliferation*						
RBC2 vs. NCT (control)	6	9	15	0	0	465
RBC2 vs. NCT (hot)	13	16	29	0	0	715
RBC2 (control vs. hot)	8	3	11	0	0	352
NCT (control vs. hot)	11	5	16	0	0	433
NCT (control vs. cold)	18	6	24	0	0	554
*Differentiation*						
RBC2 vs. NCT (control)	4	4	8	0	0	176
RBC2 vs. NCT (cold)	11	21	32	0	0	698
RBC2 vs. NCT (hot)	5	8	13	0	0	190
RBC2 (control vs. hot)	17	9	26	1	2	574
NCT (control vs. hot)	20	15	35	3	0	673
RBC2 (control vs. cold)	12	17	29	0	0	674
NCT (control vs. cold)	6	18	24	2	0	570
Totals	131	128	262	6	2	

^a^ treatment comparison name; the control group will always be either RBC2 in a genetic line comparison or control in a thermal comparison.

^b^ number of circRNAs, upregulated in the experimental treatment.

^c^ number of circRNAs, downregulated in the experimental treatment.

^d^ number of circRNAs, differentially spliced towards an increase in circular form of the transcript.

^e^ number of circRNAs, differentially spliced towards an increase in linear form of the transcript.

^f^ number of predicted miRNA, target binding sites for DECs, within that treatment; miRNA, data used were from the same biosource that were collected in Reed et al., 2023.

##### 3.3.1.1 Proliferation cell stage

Five treatment comparisons were made with SC in the proliferation stage. Across all comparisons, 62 DECs were predicted (Table 2). Three comparisons were made between thermal treatments (control 38°C vs. hot 43°C; control 38°C vs. cold 33°C) within a genetic line (RBC2; NCT) and two comparisons were made between genetic lines (RBC2 vs. NCT) within thermal treatments (control 38°C; hot 43°C). Due to no survival of RBC2-cold treated SCs, no control 38°C vs. cold 33°C within the RBC2 line or cold 33°C RBC2 vs. NCT comparisons were conducted.

###### 3.3.1.1.1 Proliferation: effects of temperature

**FIGURE 2:**
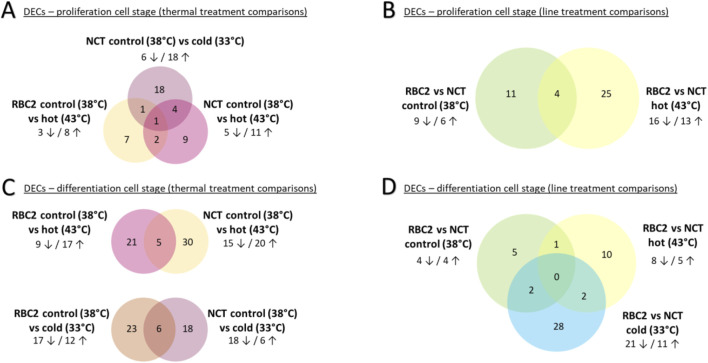
Venn diagram of DECS by treatment comparisons: **(A, C)** contain thermal treatment comparisons split by cell stages; **(B, D)** contain line treatment comparisons split by cell stages. Number of DECs given in venn diagram. Number of DECs up/downregulated within treatement comparisons are shown outside of the venn diagram.

Forty-two circRNAs were differentially expressed in comparisons between the thermal treatments within a genetic line (Figure 2A; Table 2; Supplementary Table S3) with eight circRNAs being differentially expressed in more than one treatment comparison (circ08490; circ09370; circ03849; circ01448; circ06785; circ08181; circ08945; circ10649). Two of these (circ08490, circ09370) had parent genes corresponding to known muscle protein genes, myosin light chain 10 and myogenin, respectively (Supplementary Table S4). The former being calcium binding and the latter a muscle-specific transcriptional activator that is required for skeletal muscle differentiation into multinucleated myotubes.

Heat treatment of the SCs resulted in differential expression of circRNAs in both the RBC2 and NCT cells. In the RBC2-control vs. hot comparison, 11 circRNAs were differentially expressed with three DECs being downregulated and eight upregulated at 43°C (Figure 2A; Table 2). Similarly, in the NCT-control vs. hot comparison, 26 circRNAs were differentially expressed with five DECs downregulated and 11 upregulated at 43°C. Three DECs were common to both the RBC2 and NCT control vs. hot treatment comparisons (circ08490, circ09370, and circ08945) and each were upregulated at 43°C.

In the NCT-control vs. cold comparison, 24 DECs were predicted, six being downregulated and 18 upregulated at 33°C (Figure 2A; Table 2). Five of the DECs found in the NCT-cold treatment were also DECs in the NCT-hot treatment; four were upregulated (circ01448, circ06785, circ08181, and circ08945) and one downregulated (circ03849) in both comparisons. Two DECs were shared between the in NCT control vs. cold and RBC2 control vs. hot comparisons. One (circ10649 common to all comparisons) was upregulated in NCT SCs at 33°C and downregulated in RBC2 SCs at 43°C.

###### 3.3.1.1.2 Proliferation: effects of genetic line

Forty circRNAs were differentially expressed in comparisons between the genetic lines within a thermal treatment (Figure 2B; Table 2; Supplementary Table S3). In the control temperature comparison (38°C), 15 circRNAs were differentially expressed with six DECs being upregulated and nine downregulated in the NCT line relative to the RBC2 line (Table 2; Figure 2B). In the heat treatment comparison (43°C), 29 circRNAs were differentially expressed with 13 DECs upregulated and 16 downregulated and in the NCT line relative to RBC2 line (Table 2; Figure 2B). Four DECs (circ09900, circ03853, circ03880, circ05033) were downregulated in both the control and heat treatment comparisons (Figure 2B).

##### 3.3.1.2 Differentiation cell stage

Seven comparisons were made among treatment groups for SCs in the differentiation stage. Across all comparisons, 107 circRNAs were differentially expressed. Four comparisons were made between thermal treatments (control 38°C vs. hot 43°C; control 38°C vs. cold 33°C) within genetic line (RBC2; NCT) and three comparisons were made between genetic line (RBC2 vs. NCT) within a thermal treatment (control 38°C; hot 43°C; cold 33°C).

###### 3.3.1.2.1 Differentiation: effects of temperature

Eighty-three circRNAs were found to be differentially expressed between the thermal treatments within a genetic line comparisons with 29 circRNAs being differentially expressed in more than one treatment comparison (Figure 2C; Table 2; Supplementary Table S3). In the RBC2-control vs. hot comparison, 26 circRNAs were differentially expressed with nine DECs being downregulated and 17 DECs upregulated at 43°C. In the NCT SCs, 35 circRNAs were differentially expressed with 15 DECs being downregulated and 20 upregulated at 43°C in comparison to control. Five DECs were shared between the lines; two DECs (circ04456, circ04500) were downregulated in SCs of both lines, two (circ08896, circ09289) were upregulated in both comparisons, and one (circ09901) was upregulated in the NCT but downregulated in the RBC2 cells.

In the RBC2-control vs. cold comparison, 29 circRNAs were differentially expressed with 17 DECs being downregulated and 12 DECs upregulated at 33°C (Figure 2C; Table 2; Supplementary Table S3). In the NCT-control vs. cold comparison, 24 circRNAs were differentially expressed with 18 DECs being downregulated and six upregulated in the cold treated cells in comparison to control. Six DECs were shared between the lines. Five DECs (circ06442, circ04456, circ04500, circ08492, circ08490) were downregulated at 33°C in both the RBC2 and NCT comparisons. One DEC (circ07296) was downregulated at 33°C in the NCT SCs but upregulated in the RBC2 SCs.

###### 3.3.1.2.2 Differentiation: effects of genetic line

Forty-eight circRNAs were differentially expressed between the genetic lines within thermal treatment comparisons (Figure 2D; Table 2; Supplementary Table S3). In the NCT-RBC2 comparison at control temperature (38°C) eight circRNAs were differentially expressed with four DECs being downregulated and four upregulated in the NCT SCs compared to RBC2 (Figure 2D). In the heat temperature comparison (43°C), 13 circRNAs were differentially expressed with eight DECs being downregulated and five upregulated in the NCT line in comparison to the RBC2. In the cold temperature comparison (33°C), 32 circRNAs were differentially expressed with 21 DECs being downregulated and 11 upregulated in the NCT line in comparison to RBC2 (Table 2; Supplementary Table S3).

No DECs were shared across all genetic line comparisons but five DECs (circ0991, circ07152, circ07296, circ10074, circ08181) were shared between multiple within-line comparisons. Two DECs (circ07152, circ07296) were shared between the control and cold temperature comparison, and both were downregulated in the NCT line at 33°C but upregulated in the NCT at 38°C. A single DEC (circ09901) was shared between the hot and control temperature comparisons and was upregulated at 43°C but downregulated line at 38°C in the NCT SCs compared to RBC2 cells. Of the two DECs shared between the hot and cold temperature comparisons, circ10074 was upregulated at 43°C and downregulated at 33°C in the NCT line relative to RBC2 line and circ08181 was downregulate at 43°C and upregulated at 33°C in the NCT line relative to RBC2 line (Table 2).

#### 3.3.2 Differential circRNA splicing (circRNA vs. linear forms)

Six DECs were found in three treatment comparisons to be differentially spliced (splice score > |.10|). All differentially spliced circRNAs (DSCs) occurred in the differentiating SCs and none were predicted in the proliferation SC comparisons (Table 2; Supplementary Table S3). Two circRNAs (circ04173; circ08492) were predicted to be down-spliced (increase in splicing towards the linear form) and one (circ01428) was up-spliced (increase in splicing towards the circular form) in the RBC2 control vs. hot treatment comparison. Three circRNAs (circ09737; circ06985; circ04168) were predicted to be up-spliced in the NCT control vs. hot treatment comparison and two circRNAs (circ04173; circ09737) were found to be predicted to be up-spliced in the NCT control vs. cold treatment comparisons (Table 2; Supplementary Table S3). Two DSCs (circ08492 and circ06985) have parent genes associated with known muscle genes, myosin light chain 10 and tropomyosin 1, respectively (Supplementary Table S4). The latter associated with actin-binding in contractile function. Three DSCs (circ04173, circ04168, circ04173) have the same parent gene, eukaryotic translation initiation factor 4 gamma 2 (eIF4G2, ENSMGAG00000005834). This gene employs various translational mechanisms to control gene expression involved in apoptosis, cell differentiation and survival, and embryonic development (Liu et al., 2023).

### 3.4 Functional prediction analyses

#### 3.4.1 miRNA/circRNA interaction

A total of 3,396 miRNA target binding sites (alignment score >165) corresponding to 277 turkey miRNAs was predicted for the 136 DECs with miRanda (Supplementary Table S5). For each circRNA, the miRNA with the highest alignment score (average 177.63) is given in Supplementary Table S5. Four circRNAs (circ09737; circ09900; circ09901; circ01223) had no predicted miRNA target binding sites. The number of targets per DEC ranged from 1 to 115 with an average of 24.97/circRNA. In some cases, miRNAs were predicted to bind multiple times to a single circRNA. For example, mga-miR-103 had 54 predicted target binding sites. The highest overall alignment score (200) occurred for a target binding site in circ05242 corresponding to mga-miR-26a.

Of the 277 miRNAs with target binding sites in the DECs, 43 were differentially expressed microRNAs (DEMs) in either the SC proliferation or differentiation assays (Reed et al., 2023). Eight of these miRNAs (mga-miR-206, mga-miR-2954, mga-miR-3529, mga-miR-N54, mga-miR-N40, mga-miR-N72, mga-miR-N92, and mga-miR-N185) were differentially expressed in both proliferating and differentiating SCs and had binding sites within the differentially expressed circRNAs (DECs) observed in both of the SC developmental stages.

#### 3.4.2 Secondary structure

**FIGURE 3:**
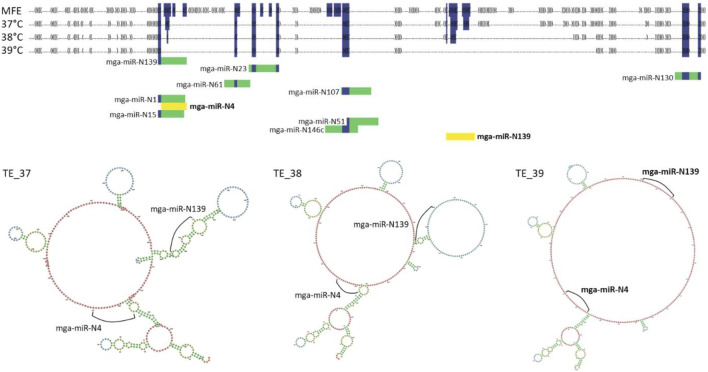
Dot-bracket secondary structure predictions for MFE and TE shown for circ09201 with miRNA target predcitions. miRNAs in yellow show target binding sites where the miRNA could bind. Folding structure predictions also shown by the 3 TE structures.3.

RNAfold was used to demonstrate the potential effect of secondary structure on miRNA/circRNA interactions. Four secondary structures were predicted and examined for circ09021 (sequence data can be found in Supplementary Table S6). Included are the minimum free energy (MFE) structure, a predicted structure that does not take temperature into account and was predicted to be the same across all three temperature parameters, and three unique predicted thermodynamic ensemble (TE) structures corresponding to 37, 38°C and 39°C (TE_37, TE_38, TE_39; Supplementary Table S6; Figure 3). In general, the higher the temperature parameter, the greater the number of predicted unpaired nucleotides (Supplementary Table S6; Figure 3). Within circ09021, 11 miRNA target binding sites were predicted (alignment score >150) using miRanda. All of the predicted target binding locations were found to be unavailable for binding in the TE_37 and TE_38 structures due to loop formations. However, the TE_39 predicted secondary structure had two target binding sites exposed for mga-miR-N4 and mga-miR-N139 demonstrating the potential effect of slight temperature change on binding interactions. To investigate how slight the temperature change needed to induce a change in miRNA binding availability, RNAfold was reran to predict the secondary structure of circ09021 at 0.1°C increment changes from 38°C to 38.5°C (Supplementary Figure S1). At 38.4°C, mga-miR-N4 was able to first bind to circ09021. Three other binding sites were made available between 38°C and 38.5°C.

## 4 Discussion

CircRNAs are naturally occurring, single-stranded RNAs formed by altered transcript splicing and are extensively expressed in eukaryotes (Drula, Braicu, and Berindan-Neagoe, 2024). Phylogenetic sequence comparisons reveal circRNAs to be moderately conserved (Patop, Wüst, and Kadener, 2019) and expression analyses suggest tissue- and development-specific expression patterns (Misir et al., 2022). Studies hypothesize that circRNAs have diverse functions (Zhang et al., 2018) such as acting as miRNA sponges, binding endogenous competing RNAs, interacting with RNA binding proteins and/or mRNAs to regulate transcription, or influencing alternative splicing (Huang Anqing et al., 2020). Several studies indicate that circRNAs play crucial roles in processes such as cell proliferation, differentiation, autophagy, apoptosis (Cao et al., 2018; Kulcheski et al., 2016; Liang et al., 2017; Panda et al., 2017), and influence gene expression during myogenesis (Das et al., 2020; Legnini et al., 2017). Results from this study further support the hypothesis that differential expression of circRNAs are impacted by genetic background and changes in temperature in turkey muscle SCs.

### 4.1 circRNA prediction and quantification

The number of unique circRNAs predicted from RNAseq analyses in poultry, varies greatly among studies. Analysis of breast muscle from embryonic chickens of multiple embryonic days, identified 4,226 circRNAs (Shen Xiaoxu et al., 2019) and over 13,000 unique circRNAs were identified from embryonic skeletal muscle (Ouyang et al., 2018). The circRNAs identified here in the turkey SCs (10,679) was greater than the number identified (8,924) in our analysis of skeletal muscle from thermally challenged turkey poults (Reed et al., 2021). The average length of circRNAs found in this study (average: 37,980 nt) exceeds those reported in circRNA studies conducted in chicken (average: 200–2,564 nt, range: <61–95985 nt; Chen et al., 2023; Huang Xuewei et al., 2020; Shen Manman et al., 2019; Wang et al., 2022), but falls within the range observed in our study of whole turkey muscle (average: ∼36,200 nt, range: 134–∼200,000 nt; Reed et al., 2021) and that of some mouse and human studies (average: 18,132 nt, range: 51–922930 nt; Vromman et al., 2023). The difference in average length could be a result of several factors including the use of CIRI2 compared to other prediction programs with different underlying prediction algorithms, variations in genome quality, differences in sequence read length, and alternative splicing events. A cursory analysis of the SC sequences with CIRI-AS, a program that detects alternative splicing of circRNAs, indicates occurrence of alternative splicing events. However, due to the limited sensitivity of read lengths <75 nt, further investigation was not pursued.

The circRNAs predicted in the turkey SCs were distributed across the genome. To investigate if there was a skewed distribution of circRNA prediction among chromosomes, a chi-square test between number of circRNAs predicted per chromosome and length of chromosome was conducted. A *p*-value of 0.24 indicates that the number of predicted circRNAs is most likely due to the chromosome length and no bias in chromosome representation was observed. The differential expression of circRNAs from muscle-related parent genes is not unexpected given that the source of the RNAseq data is muscle stem cells.

### 4.2 RNase depletion

Although most circRNAs occur at comparatively low levels, some surpass the expression levels of their linear RNA counterparts (Wilusz, 2018). Due to their resistance to degradation by RNA exonuclease, circRNAs are more stable than linear RNAs. RNase R treatments are commonly used to degrade linear RNA for circRNA enrichment. This enrichment allows for direct comparison of circRNA predictions from sequence data with and without enrichment. Although some characteristics such as genomic distribution were similar between the matched sources, RNase depletion of the SC RNAs resulted in a 50%–80% reduction in the number of circRNAs predicted, and detection of 2,775 of previously undetected circRNAs in the non-depleted libraries. This highlights a potential weakness of data mining traditional mRNA data sets *versus* broader use of RNase depletion in library preparation and sequence generation.

### 4.3 Differential expression

This study of skeletal muscle SCs suggests that the production of circRNAs is significantly altered by thermal challenge as predicted in the muscle of thermally challenged turkey poults (Reed et al., 2021). Of the 140 DECs identified in the turkey SC’s, 137 were also identified in our earlier study of muscle from turkey poults (Reed et al., 2021) suggesting conserved expression. Ten of these circRNAs (circ01223, circ01670, circ01781, circ01919, circ04932, circ05562, circ07670, circ08490, circ09289 and circ10481) were differentially expressed in both studies (Supplementary Table S7).

#### 4.3.1 Thermal treatment comparisons

In all comparisons, the number of circRNAs showing increased expression was higher in cells incubated at the higher temperature. Temperature is a key regulatory factor for alternative mRNA splicing in both plants and animals (Shiina and Shimizu, 2020). Temperature-dependent alternative mRNA splicing has been demonstrated in mammals as a response to low body temperature during hibernation (Sano et al., 2015). Other effects on RNA splicing related to cellular temperature are changes in transcription rate of RNA polymerase II and RNA structure (Capovilla et al., 2015). Modifications of secondary RNA structure can affect the positioning of the spliceosomal complex which catalyzes backsplicing for the creation of circRNAs (Shi et al., 2021). These factors might contribute to the differences seen in circRNA expression in the cultured SCs. The differential expression of circRNAs with parent genes corresponding to known muscle protein genes demonstrates this potential impact. The cellular mechanisms of temperature-dependent alternative splicing continue to be the focus of ongoing research. Another potential cause to the differences in circRNA expression is that some of the circRNAs identified could simply be artifacts, though CIRIquant incorporates a multiple seed matching to help eliminate false positives (Drula et al., 2024; Gao et al., 2018)

The potential for shifts in gene expression due to thermal stress is important in poultry biology and ultimately meat production. Birds are homotherms but young birds have poor thermal regulation and they cannot maintain their body temperature when under even relatively mild thermal stress (Henrikson et al., 2018; Meltzer, 1983; Yahav, 2000). Thermal stress especially after hatch can in turn have long-term effects on the growth and structure of muscle (Patael et al., 2019; Piestun et al., 2017). Timing and degree of thermal stress are critical. Although development of satellite cells can be thermally stimulated during specific times in embryonic development, studies highlight changes in breast muscle histomorphology, metabolism, and physiology resulting from suboptimal incubation conditions (Oviedo-Rondón et al., 2020). For example, in broilers, mild thermal manipulation during early posthatch period can promote myogenic proliferation and differentiation (Halevy, 2020), whereas heat stress during the first 13 D posthatch can increase fat and collagen deposition and impair growth of the pectoralis muscle (Patael et al., 2019). Birds in production systems face potential new challenges due to climate change. These include not only higher mean temperatures, but also the increased frequency of extreme weather events, both hot and cold (Ruuskanen, Hsu, and Nord, 2021), which may in turn increase the incidence of adverse effects on muscle growth, development, and ultimately on meat quality.

#### 4.3.2 Genetic line comparisons

Thermal comparisons within genetic lines (NCT and RBC2) found the majority of DECs were unique to genetic background with few DECs shared between lines in both proliferating and differentiating SCs (Supplementary Table S3). Differences in the DECs shared between the NCT and RBC2 lines can be attributed to underlying genetic differences from selection for commercial traits in the NCT birds. The RBC2 line been maintained at The Ohio State University, Poultry Research Center (Wooster, OH) without conscious selection for any trait since 1966 and has significantly different growth characteristics than the modern commercial bird (Lilburn and Nestor, 1991).

### 4.4 Functional predictions

#### 4.4.1 circRNA/miRNA interactions

There is increasing evidence for the importance of RNA interactions in muscle biology. For example, our recent examination of miRNA expression in turkey muscles SCs in this same experimental system (Reed et al., 2023), detected significant expression differences among temperature treatments and between genetic lines. Including the eight miRNAs that had target binding sites with the DECs found in this study. In both proliferating and differentiating turkey SCs, significant differential expression of miRNAs important in muscle development (e.g., mga-miR-206) was found in response to thermal challenges in both turkey lines. Studies by Harding and Velleman (2016), and by Velleman and Harding (2017), suggest that miRNAs play roles in myogenic satellite cell migration. Interactions between miRNAs and circRNAs have the potential to alter these processes (Liang et al., 2017; Ouyang et al., 2018). These studies in turkey demonstrate how changes in non-coding RNAs could significantly affect cellular proliferation and differentiation, potentially affecting *Pectoralis major* muscle growth and development and subsequently breast meat quality.

Predictions of circRNA-miRNA interaction are often used support the hypothesis that circRNAs function as “molecular sponges”. As demonstrated here, miRanda and related programs can identify thousands of potential miRNA target binding sites associated with circRNA sequences. However, identification of potential interactions does not translate directly to downstream function and detailed biochemical experiments are necessary to verify predicted interactions. The circRNAs identified in this study and the predicted miRNA interactions provide a framework for generating future hypotheses.

#### 4.4.2 Secondary structures

Previous examinations of circRNAs have not considered how the cellular environment likely affects the function of these RNA molecules. As single stranded RNAs, circRNAs would create complex secondary and tertiary structures as a result of RNA folding. These dynamic structures respond to factors such as pH and temperature. As we have shown, a temperature increase of as little as 0.4°C induced changes in predicted secondary structure and accessibility of miRNA target binding sites (Supplementary Figure S1). These predicted changes in binding ability due to RNA structures are likely what is happening biologically within cells suggesting that even slight changes in body temperature may increase or decrease miRNA binding or binding of circRNAs to proteins. While miRNA/circRNA interactions are important to understand modulation of gene expression, changes in secondary and tertiary structures from temperature changes should also be considered important (Somero, 2018). To the best of our knowledge, this is the first time miRNA/circRNA interactions have been viewed through the lens of secondary structure.

## 5 Conclusion

This investigation identified and quantified circRNAs expressed in turkey skeletal muscle stem cells cultured from the *Pectoralis major* muscle, under thermal challenge. The expression of circRNAs was significantly influenced by thermal treatments and the genetic background of the stem cells, with differentially expressed circRNAs (DECs) identified in each comparison. While the potential for these DECs to interact with miRNA targets is predicated to be significant, RNA secondary structure must be considered when evaluating these potential interactions. It is crucial to note that our circRNAs predictions and their interactions with miRNAs are theoretical and the circRNAs identified in this study were not experimentally verified. While these predictions offer hypotheses and a foundation for future research into the underlying cellular mechanisms, additional verification is required. The impact of environmental stressors on non-coding RNAs and their role in gene regulation remains a critical area of research, directly connected to muscle development and poultry production.

## Figures and Tables

**Table:** 

**DEC(s)**	differentially expressed circular RNA(s)
**DEM(s)**	differentially expressed microRNA(s)
**DS**	differential splicing
**DSC(s)**	differentially spliced circRNA(s)
**SC(s)**	satellite cell(s)
**NCT**	Nicholas Commercial Turkeys
**RBC2**	Randombred Control Line 2

## Data Availability

Publicly available datasets were analyzed in this study. This data can be found here: SRA BioProject PRJNA842679.
